# The Role of the Subgenual Anterior Cingulate Cortex and Amygdala in Environmental Sensitivity to Infant Crying

**DOI:** 10.1371/journal.pone.0161181

**Published:** 2016-08-25

**Authors:** Isabella Mutschler, Tonio Ball, Ursula Kirmse, Birgit Wieckhorst, Michael Pluess, Markus Klarhöfer, Andrea H. Meyer, Frank H. Wilhelm, Erich Seifritz

**Affiliations:** 1 Department of Psychology, Division of Clinical Psychology and Epidemiology, University of Basel, Basel, Switzerland; 2 Department of Psychological Sciences, University of San Diego, San Diego, United States of America; 3 Bernstein Center Freiburg, University of Freiburg, Freiburg, Germany; 4 Intracranial EEG and Functional Brain Imaging Research Group, University of Freiburg, Freiburg, Germany; 5 Department of Psychology, Division of General and Biological Psychology, University of Konstanz, Konstanz, Germany; 6 Department of Biological and Experimental Psychology, School of Biological and Chemical Sciences, Queen Mary University of London, London, United Kingdom; 7 MR-Physics, University Hospital Basel, Basel, Switzerland; 8 Division of Clinical Psychology, Psychotherapy, and Health Psychology, Department of Psychology, University of Salzburg, Salzburg, Austria; 9 Department of Psychiatry, Psychotherapy and Psychosomatics, Psychiatric Hospital, University of Zurich, Zürich, Switzerland; Western University, CANADA

## Abstract

Newborns and infants communicate their needs and physiological states through crying and emotional facial expressions. Little is known about individual differences in responding to infant crying. Several theories suggest that people vary in their environmental sensitivity with some responding generally more and some generally less to environmental stimuli. Such differences in environmental sensitivity have been associated with personality traits, including *neuroticism*. This study investigated whether neuroticism impacts neuronal, physiological, and emotional responses to infant crying by investigating blood-oxygenation-level dependent (BOLD) responses using functional magnetic resonance imaging (fMRI) in a large sample of healthy women (N = 102) with simultaneous skin conductance recordings. Participants were repeatedly exposed to a video clip that showed crying infants and emotional responses (valence, arousal, and irritation) were assessed after every video clip presentation. Increased BOLD signal during the perception of crying infants was found in brain regions that are associated with emotional responding, the amygdala and anterior insula. Significant BOLD signal decrements (i.e., habituation) were found in the fusiform gyrus, middle temporal gyrus, superior temporal gyrus, Broca’s homologue on the right hemisphere, (laterobasal) amygdala, and hippocampus. Individuals with high neuroticism showed stronger activation in the amygdala and subgenual anterior cingulate cortex (sgACC) when exposed to infant crying compared to individuals with low neuroticism. In contrast to our prediction we found no evidence that neuroticism impacts fMRI-based measures of habituation. Individuals with high neuroticism showed elevated skin conductance responses, experienced more irritation, and perceived infant crying as more unpleasant. The results support the hypothesis that individuals high in neuroticism are more emotionally responsive, experience more negative emotions, and may show enhanced cognitive control during the exposure to infant distress, which may impact infant-directed behavior.

## Introduction

Infant crying is considered to be a powerful communication signal ensuring infants`survival [1] and it includes both facial and vocal components [2]. Infant crying signals evoke strong emotional reactions, ranging from empathy to distress [3]. It has been shown that infant crying can also trigger child abuse such as neglect, slapping, and shaking [4], therefore, it is important to understand the emotional responses and the neural structures that mediate adults`emotional reactions to infant crying. Studies that investigated the neural responses to infant distress find circuits that include activation in the amygdala, anterior insular cortex, and inferior frontal gyrus [5–7]. However, little is known about individual differences in responding to infant crying. Several theories suggest that people vary in their environmental sensitivity with some responding generally more and some generally less to environmental stimuli [8–10]. Such differences in environmental sensitivity have been associated with a range of genetic, physiologic and behavioral factors, including the personality trait *Sensory-Processing Sensitivity* [11] that correlates with neuroticism (r = .40, see [12]), one of the five empirically derived main personality traits (“Big-5”). Neuroticism is characterized by the tendency to experience negative affect and distress [13] and hence, may be of particular interest in relation to how individuals respond to infant crying. Previous work has shown that individuals scoring high in neuroticism exhibit elevated skin conductance reactivity in response to emotionally distressing events when compared to emotionally more stable individuals [14]. High neuroticism appears to confer stress vulnerability when situations are perceived as threatening [15]. Neuroticism affects emotion regulation, which is the ability to control an emotion and the active attempt to modify a negative emotion towards a more positive emotional state [16]. Neuroticism has been associated with the amygdala and subgenual anterior cingulate cortex (sgACC) [17]. Research shows that these regions are functionally coupled [18] and that individuals with lesions in the sgACC region demonstrated abnormal autonomic responses during emotion processing [19]. More recently it was been shown that the sgACC is involved in overcoming a real-life stressful situation [20] and may thus play an important role when individuals with high neuroticism are repeatedly exposed to infant distress.

Previous studies underscores the importance of investigating temporal dynamics when studying affective processing using neuroimaging methods [21,22]. Studies in humans using various neuroimaging techniques such as functional magnetic resonance imaging (fMRI) [21,23] and magnetoencephalography (MEG) [24] have shown that brain areas *habituate* to repeated stimuli presentations—that is brain responses decline upon repeated stimulus presentations. Research has shown that habituation in response to repeatedly presented emotional stimuli include the (laterobasal) amygdala [21,23,25]

This study investigated whether neuroticism impacts neuronal, physiological and emotional responses to infant crying in healthy women using fMRI with simultaneous skin conductance recordings. This study repeatedly presented a video clip that showed crying infants. Participants`emotional responses were assessed by immediate ratings of valence, arousal, and irritation after every video clip presentation. Valence and arousal are considered to be two basic dimensions of emotional experience [26]. A study by Riem and colleagues found that infant crying also evokes feelings of irritation [27]. Furthermore, Seifritz and colleagues demonstrated that parents showed more amygdala activation than nonparents, suggesting that neuronal responses to infant crying may be modulated by parenting experiences [28]. It has been shown that sex differences exist in response to a baby crying [29]. Consequently, in order to account for such confounding influences, we recruited a large sample of healthy women (N = 102) that had no children of their own and *no* experience in professional child care, and investigated the following hypotheses: First, we expected that the exposure to infants crying evokes activation in brain regions that are associated with emotional responding. Second, we expected habituation of the blood-oxygenation-level dependent (BOLD) signal during the repeated exposure to infant crying in cortical and subcortical brain regions such as in the laterobasal amygdala. Third, we hypothesized that high-neuroticism individuals show during the repeated exposure to infant crying more activation in sgACC, which is involved in cognitive control during distressing emotions. Fourth, we expected that individuals with high neuroticism experience more negative emotions (assessed by valence and irritation ratings after every video presentation), exhibit greater skin conductance responses, and show greater amygdala activation during infant crying, because they are emotionally more responsive. Finally, we hypothesized that women high in neuroticism demonstrate less habituation on the neuronal, the physiological (measured by skin conductance recordings), as well as on the emotional level (assessed by valence, arousal, and emotional irritation ratings after every video presentation) in response to repeated infant crying.

## Method

### Participants

One-hundred-and-two healthy women were recruited from the University of Basel, Switzerland and participated in the fMRI experiment (mean age = 23.64, range = 18–35 years). Five participants were excluded from fMRI data analysis because of excessive motion (head movement exceeded 1.0 mm in any of the x, y, and z directions). Three participants were excluded from the analysis of skin conductance responses because of technical difficulties during recoding. Psychological measures and subjective affective ratings were analyzed from all individuals. Participants were screened for *exclusion criteria* using a checklist, comprising the following criteria: (1) Participants with children and experience in professional childcare at the time of investigation, (2) a history of seizure or head injury, (3) the use of medication that can influence the test results, (3) visual or auditory problems that cannot be corrected, (4) MRI incompatible implants, (5) pregnancy, (6) claustrophobia, (7) a history of psychiatric or neurological disorders. The ethics committee for medical research in Basel, Switzerland approved the study. Before participation, subjects gave their written informed consent.

### Procedure

Participants were given 30 min to adjust to the laboratory setting. During this time they received instructions for the experiment. Subsequently, subjects were placed in the MR scanner and were asked to complete the state anxiety scale with 20 items [30] by using an MR-compatible mouse (please note: state-anxiety findings are reported elsewhere in relation to genetic polymorphisms [31]). Subsequently, 43 video clips were presented using stimulus presentation software (E-Prime 2.0, Psychology Software Tools, Sharpsburg, PA, USA). Video clips were presented via an LCD beamer that projected to a screen positioned behind the participants, which was visible over a mirror mounted on the head coil. Audio was presented via magnetic resonance compatible headphones (Resonance Technology, Los Angeles, USA). Subjects were instructed to attend to the video clips and to avoid any movement. The experiment had three phases: First, participants were *familiarized* with the experimental setting with a video clip that showed laughing infants (LI). That video clip was shown five times. Subsequently, a video clip with crying infants (CI) was presented 33 times including three video clip presentations that were varied for an attention-control task (please note: the findings related to that attention-control task are presented elsewhere [31]). This phase was used to assess *habituation*. At the end of the experiment we presented a video clip that showed laughing infants (i.e., another emotional category than during the habituation phase). This video clip was shown five times. This phase was used to distinguish habituation from sensory and motor fatigue [32]. Participants were asked to evaluate every video clip on a seven-point bipolar scale along the dimensions valence (ranging from –3 = unpleasant to 3 = pleasant), arousal (ranging from –3 = calming to 3 = arousing), and irritation (ranging from –3 = relaxed to 3 = irritated). Each evaluation period lasted 18 s. Participants conveyed what they felt by using a mouse that allowed them to move a white box on the visually presented scale leftwards or rightwards by pressing the left and right mouse buttons with their right hand. Each evaluation was followed by a baseline time window of 15 s duration (i.e., when subjects passively viewed a fixation cross) used as baseline for the fMRI. In total, there were 43 runs (each consisting of video presentation, evaluation, and baseline), each with a 60 s duration. After these 43 runs, the state-anxiety questionnaire was completed a second time in the scanner under the same conditions. The total duration of scanning was approximately 55 min per session. The experiment is illustrated in Fig A in S1 File.

### Questionnaires

To asses neuroticism, all participants completed the German version of the *NEO Five Factor Inventory* [13]. This questionnaire consists of 60 items and measures five different personality traits: neuroticism, extraversion, openness, agreeableness and conscientiousness Participants also completed the *Center for Epidemiologic Studies Depression Scale* (CES-D) [33] which assesses depressive symptoms. The CES-D is a 20-item measure that asks individuals how often over the past week they experienced symptoms associated with depression, such as restless sleep, poor appetite, and feeling lonely. Handedness was assessed with a questionnaire by Oldfield [34].

### Acquisition of skin conductance responses (SCRs)

Skin conductance was continuously recorded from the thenar and hypothenar palm of the immobilized, left hand in parallel to the fMRI recording. Data were saved at a sampling rate of 200 Hz using the BIOPAC MP150 system skin conductance module (Biopac Systems, Inc., Goleta, California, USA) with MR-conditional, disposable electrodes (EL509) filled with isotonic gel (TD-246, Med Associates paste). Electrode cables were grounded and passed through an RF filter panel and stimulus timing information was recorded via TTL pulses from the MR scanner to synchronize fMRI and physiological data analysis.

### Acquisition of fMRI data

Functional images were acquired on a 3T scanner (Siemens Magnetom Allegra MR 2004A, Erlangen, Germany). Image acquisition started with a localizer, a reference scan for the distortion correction, and a magnetization-prepared rapid-acquisition gradient echo (MPRAGE) sequence of 7-min duration (resolution: 1 mm x 1 mm x 1 mm, matrix: 256 * 256 * 176, TR: 2000 ms, TI: 1000 ms, 7° flip angle). Functional images were obtained using a multislice gradient echo planar imaging (EPI) method. Each volume consisted of 44 transversal slices (2.5 mm slice thickness with a 0.5-mm interslice distance, matrix: 96 * 96, field of view 240 mm * 240 mm resulting in 2.5 mm x 2.5 mm x 2.5 mm resolution, repetition time (TR) 3000 ms, echo time (TE) 35 ms, 90° flip angle). An accurate registration of the functional images was accomplished by online correction of the functional image data for geometric distortions [35]. The distortion field was derived from the local point-spread function (PSF) in each voxel as determined in a one-minute reference scan (see above). Prior to distortion correction, data were online motion-corrected by image realignment to the reference scan. A representative example of functional images showing the amygdala after application of the distortion correction algorithm is shown in the study by Ball et al., [36].

### Data Analysis

#### Preprocessing and statistical analysis of fMRI data

Data analysis was performed using SPM5/12 (http://www.fil.ion.ucl.ac.uk/spm/). Preprocessing consisted of realignment, and normalization, and smoothing. All functional images were normalized into standard stereotaxic space of the Montreal Neurological Institute (MNI) template. The images were smoothed using a 9 mm full-width-at-half-maximum (FWHM) Gaussian kernel to minimize the effects of individual variations in anatomy and to improve the signal-to-noise ratio. A high-pass filter with a cut-off at 1/128 Hz was applied before parameter estimation. **First level modeling**: Regressors were the timing information of the video clips and the timing information of the evaluation period after every video clip presentation. Regressors were modeled with a boxcar function convolved with a canonical hemodynamic response function. The first model included the following regressors: 9 for the video clips (LI at the beginning and at the end, 6 for CI because the 30 crying-infant video clip presentations were grouped into 6 consecutive blocks, and 1 for the control film) and one for the evaluation period after each film clip. Additional regressors were included to model the six head-movement parameters obtained during realignment. For each individual a contrast image *crying infants>baseline* (that is the time periods during which subjects passively viewed the fixation cross without stimulus presentation) was analyzed. To analyze response decrements the 30 crying-infant video clip presentations were grouped into 6 consecutive blocks and *habituation* was modeled as a linear change across these groups (duration 35 min). The attention-control variant of the CI video was not included in the statistical analyses. **Second level group analyses:** Brain areas with significant response decrements (*habituation*) during crying-infant presentations were tested at p < 0.05, family wise error (FWE) corrected for multiple comparisons, cluster size ≥ 15 voxels. The contrast perception of *crying infants > baseline* was tested at p < 0.05, FWE corrected, cluster size ≥ 15 voxels. To test whether individuals with high neuroticism differ in comparison to individuals with low neuroticism in terms of amygdala and sgACC activation a two-sample t-test was applied and tested at p < 0.05, FWE small volume correction. A small volume correction was performed because the amygdala and sgACC were included in our a *priori* hypothesis based on previous research showing that both brain regions are associated with neuroticism [17]. The median (neuroticism score: 1.67) served as boundary between the high and the low neuroticism groups. A 10 mm radius sphere was placed in sgACC (MNI coordinate: 6/42/-16) and the amygdala (MNI coordinate: 22/-8/-12) based on previous findings that show the involvement of sgACC and amygdala in neuroticism [17].

#### Preprocessing of skin conductance responses

Offline data analysis of *skin conductance responses* (SCR) waveforms was conducted using ANSLAB software (http://www.sprweb.org). Recordings were visually inspected and periods of signal loss were manually excluded (approx. 1% of video epochs). For each video presentation, the level of SCR was assessed as the pre-film baseline (2 s) to peak difference for the largest deflection in the 0–15 s video window [37]; values > 0.005 μS were accepted as valid non-zero SCR responses). For between-subject standardization, SCRs exceeding 3 SD of a subject’s SCR mean were excluded and SCRs were then scaled relatively to a subject’s maximal SCR [38,39] resulting in a range of 0 to 1 for all SCR values.

#### Statistical analysis of skin conductance responses and psychological data

Statistical analyses were conducted using SPSS (Version 16.0, Chicago, Illinois, USA). For analysis of the time-course of SCR levels, as well as valence, arousal, and irritation ratings, values for five subsequent video clip presentations were averaged into one value. Thus, one mean value was obtained by averaging across the five video clip presentations with laughing infants at the beginning of the experiment (*Laugh_1*; *Familiarization*), six mean values (thirty video clips averaged across each of five) were obtained with crying children (*Cry_1 to Cry_6*; *Habituation*), and finally one mean value was obtained by averaging across five video clip presentations with laughing children presented at the end of the experiment *(Laugh_2)*. The attention-control variant of the CI video was not included in the statistical analysis. All four outcome measures were analyzed using linear mixed models and each outcome measure was checked for normality using QQ-plots. SCR data were transformed using reciprocal transformation while the three ratings were left untransformed. The mixed model for testing habituation contained neuroticism as continuous between-subjects variable and time (Cry_1 to Cry_6) as within-subjects variable. The test for fatigue at the end of the experiment included neuroticism as continuous between-subjects variable and time with the levels Cry_6 versus Laugh_2. Interaction effects between neuroticism and time were also tested but were not significantly different from 0 for any outcome measure and therefore not considered further in the analysis of the SCR and rating data. The models also included a random intercept and, if significantly improving model fit, a random linear slope for time. To test whether at the end of the experiment (block Laugh_2) psychological (arousal, valence, irritation) and physiological (SCR) data were related to subjects’ neuroticism scores, we performed linear regression models with neuroticism as predictor and the respective psychological or physiological variable as outcome.

## Results

### Functional imaging data

FMRI results revealed increased BOLD signal during the *perception of crying infants > baseline* (i.e., when subjects passively viewed a fixation cross) in the Heschl’s gyrus, visual cortex, superior temporal gyrus (STG), insular cortex, fusiform gyrus, temporal pole, inferior frontal cortex (including Broca’s area and Broca’s homologue on the right hemisphere), supplementary motor area (SMA), cerebellum, amygdala, and hippocampus (*p*<0.05, FWE corrected, clustersize ≥ 15 voxels). All brain regions, MNI coordinates, and T-scores are provided in Table 1.

**Table 1:** Significant fMRI blood-oxygen-level dependent (BOLD) signal for the contrast *perception of crying infants > baseline* (*p* < 0.05 FWE corrected, clustersize clustersize ≥ 15 voxel). Peak MNI-coordinates and T-values are given. Anatomical assignments were performed using a probabilistic anatomical atlas system [41,42].

MNI-Coordinates (x/y/z)			T-score	Brain region	Probabilistic map
10	-85	-9	22.48	Visual cortex	Area 18: 90% (assigned)
-8	-98	12	22.17	Visual cortex	Area 17: 60% (assigned)
43	-50	-24	22.09	Right fusiform gyrus	No map
-13	-75	-27	21.23	Left cerebellum	No map
15	-95	15	21.12	Visual cortex	Area 18: 70% (assigned)
48	-78	-6	20.80	Right inferior occipital gyrus	No map
18	-95	21	20.71	Visual cortex	Area 18: 50% (assigned)
50	-8	-6	20.31	Right Superior Temporal Gyrus	No map
-8	-78	-45	20.27	Left cerebellum	No map
40	-80	-15	19.38	Right inferior occipital gyrus	hoC4v (V4): 60% (assigned)
-38	-25	6	19.29	Left Heschl’s gyrus	Te 1.1: 50% (assigned)
25	-95	21	19.19	Right superior occipital gyrus	Area 18: 30%
25	-78	-12	19.17	Right fusiform gyrus	hOC3v (V3v): 50% (assigned)
-45	-13	-3	19.06	Left superior temporal gyrus	No map
-50	-78	3	18.94	Left middle occipital gyrus	No map
45	-20	6	18.94	Right Heschl’s gyrus	Te 1.1: 50% (assigned)
55	-13	3	18.82	Right superior temporal gyrus	Te 1.0: 70% (assigned)
-43	-50	-24	18.50	Left fusiform gyrus	No map
0	-90	3	18.47	Visual cortex	Area 17: 100% (assigned)
63	-28	3	18.42	Right superior temporal gyrus	No map
48	-65	3	18.42	Right middle temporal gyrus	hOC5 (V5): 60% (assigned)
68	-35	9	18.20	Right inferior parietal cortex	IPC (PF): 60% (assigned)
-38	-63	-27	17.68	Left cerebellum	No map
45	23	21	17.65	Right inferior frontal gyrus	Area 45: 10%
40	-63	-18	17.24	Right fusiform gyrus	No map
58	-68	0	17.08	Right middle temporal gyrus	hOC5 (V5): 20%
-55	-18	3	17.04	Left superior temporal gyrus	Te 1.2: 20%
-48	0	-15	16.89	Left middle temporal gyrus	No map
30	-10	-15	16.71	Right hippocampus	CA: 50% (assigned)
18	-3	-21	16.11	Right amygdala	SF: 70% (assigned)
-33	-78	-15	16.10	Left fusiform gyrus	hOC4v (V4): 40% (assigned)
-48	-68	-30	16.02	Left cerebellum	No map
-58	-38	12	16.02	Left inferior parietal cortex	IPC (PFcm): 20%
-18	-83	-12	15.86	Left linual gyrus	hoC4v (V4): 50% (assigned)
-40	-68	-18	15.76	Left fusiform gyrus	hoC4v (V4): 10%
53	-40	9	15.54	Right middle temporal gyrus	No map
-45	-70	9	15.45	Left middle temporal gyrus	hOC5 (V5): 20%
33	3	-24	15.16	Right amygdala	LB: 10%
8	-80	-42	15.06	Right cerebellum	No map
-58	-28	6	14.69	Left superior temporal gyrus	Te 1.1: 10%
40	-68	-27	14.61	Right cerebellum	No map
-18	-5	-21	14.53	Left amygdala	SF: 100% (assigned)
20	-93	30	14.41	Right superior occipital gyrus	Area 18: 20%
50	5	51	14.11	Right precentral gyrus	Area 6: 20%
55	33	18	14.11	Right inferior frontal gyrus	No map
48	13	33	13.29	Right inferior frontal gyrus	No map
-33	-10	-15	13.02	Left Hippocampus	CA: 30%
55	30	-3	12.96	Right inferior frontal gyrus	Area 45: 50% (assigned)
-10	-88	42	12.46	Left superior occipital gyrus	SPL (7P): 10%
43	30	-6	12.40	Right inferior frontal gyrus	No map
28	-83	24	12.36	Right superior occipital gyrus	No map
8	-90	30	12.23	Right cuneus	Area 18: 20%
15	8	9	12.21	Right caudate nucleus	No map
-18	-38	-48	12.11	Left cerebellum	No map
-28	-70	-48	11.75	Left cerebellum	No map
25	-58	-9	11.62	Right linual gyrus	No map
-30	10	-33	11.45	Left temporal pole	No map
40	-88	12	11.44	Right middle occipital gyrus	No map
-23	0	-30	11.16	Left amygdala	LB: 70% (assigned)
28	35	-18	11.04	Right inferior frontal gyrus	No map
-55	-58	9	10.78	Left middle temporal gyrus	IPC (PGp): 10%
-65	-20	-6	10.78	Left middle temporal gyrus	Te 3: 10%
10	-83	48	10.75	Right superior parietal lobule	SPL (7P): 50% (assigned)
33	-78	-24	10.70	Right cerebellum	No map
-50	-38	21	10.68	Left inferior parietal cortex	IPC (PFcm): 40% (assigned)
10	-13	9	10.56	Right thalamus	No map
-25	-88	-27	10.49	Left cerebellum	No map
20	-43	-12	10.27	Right fusiform gyrus	No map
0	-40	-3	9.83	Cerebellar vermis	No map
43	30	-18	9.52	Right inferior frontal gyrus	No map
30	-63	-33	9.36	Right cerebellum	No map
-28	-53	-9	9.35	Left fusiform gyrus	Area 17: 10%
65	-38	-12	9.20	Right middle temporal gyrus	No map
-18	-43	-9	9.18	Left linual gyrus	No map
25	-80	48	9.12	Right superior parietal lobule	SPL (7P): 10%
28	-3	-42	9.04	Right hippocampus	EC: 60% (assigned)
-20	-63	-12	8.65	Left linual gyrus	hoC4v (V4): 50% (assigned)
-13	-53	-42	8.37	Left cerebellum	No map
50	-60	-33	8.32	Right cerebellum	No map
40	-13	-33	7.56	Right fusiform gyrus	No map
23	-50	0	7.50	Right linual gyrus	Area 18: 50% (assigned)
38	20	57	6.87	Right middle frontal gyrus	No map
-43	13	27	10.98	Left inferior frontal gyrus	Area 44: 60% (assigned)
-50	23	24	10.68	Left inferior frontal gyrus	Area 45: 70% (assigned)
-43	3	39	8.54	Left precentral gyrus	No map
-33	21	-3	9.14	Left insula	No map
5	10	66	10.72	Right motor area	Area 6: 60% (assigned)
0	38	53	9.28	Left superior medial gyrus	No map
5	23	54	8.90	Right supplementary motor area	Area 6: 20%

**Fig 1:**
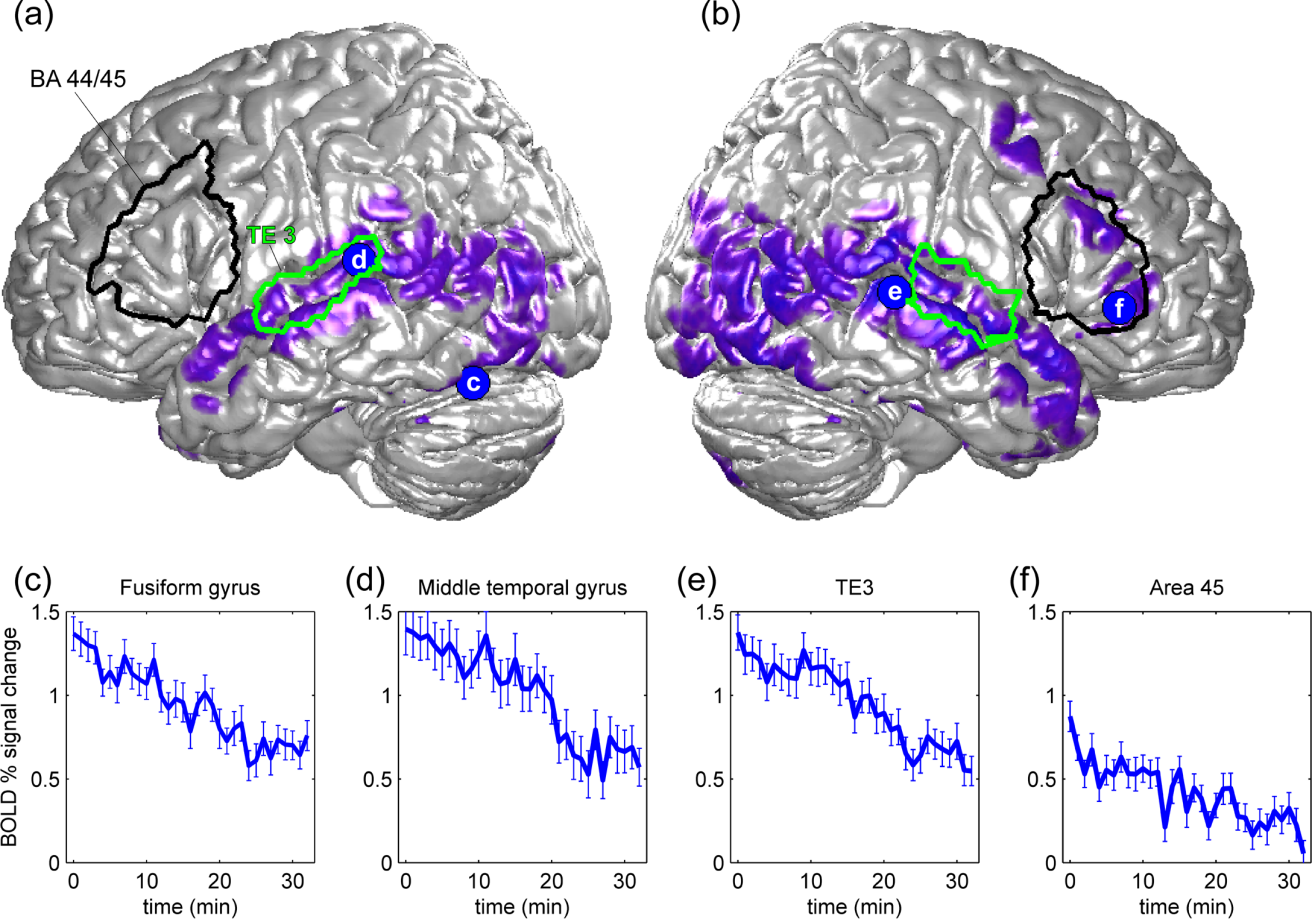
Illustrates significant fMRI blood-oxygen-level dependent (BOLD) signal decline during crying infant (CI) presentations. Significant response decrements (*p* < 0.05, FWE-corrected) during CI presentations are shown in purple and rendered on a standard brain surface, (**a**) the left and (**b**) right hemisphere. The outline of brain-surface projections of the areas BA44/45 and TE3 from a probabilistic atlas system [41,42] are indicated by black and green lines. **(c)** to **(f)**: Significant BOLD signal decline (i.e., habituation) was found in the fusiform gyrus, middle temporal gyrus, TE3 (superior temporal gyrus [40]), and in the right BA 45. Median percentage of BOLD signal change over the 30 CI presentations for the four example peaks shown in (a) and (b). Error bars indicate standard errors.

Significant fMRI BOLD signal decrements during crying infant presentations (35 min) were found in the fusiform gyrus, middle temporal gyrus, superior temporal gyrus (TE3 [40]), Broca’s homologue on the right hemisphere (BA 45), the (laterobasal) amygdala, hippocampus, and cerebellum (*p*<0.05, FWE corrected, clustersize ≥ 15 voxels, see Fig 1). Table 2 provides all brain regions, MNI-coordinates, and T-scores.

**Table 2:** Significant fMRI blood-oxygen-level dependent (BOLD) signal decline during crying infant presentations. (*p* < 0.05 FWE corrected, clustersize clustersize ≥ 15 voxel). IPC = inferior parietal cortex, hOC = human occipital lobe, Te = auditory cortex, EC = entorhinal cortex, SUB = subicular complex, LB = laterobasal amygdala, area 45 = Broca’s homologue, and area 6 = premotor cortex. Peak MNI-coordinates and T-values are given. Anatomical assignments were performed using a probabilistic anatomical atlas system [41,42]

MNI-Coordinates (x/y/z)			T-score	Brain region	Probabilistic map
45	-40	-21	11	Inferior temporal gyrus	No map
60	-32.5	0	10.8	Middle temporal gyrus	No map
65	-30	9	10.2	Inferior parietal cortex	IPC (PF): 40% (assigned)
57.5	-15	-6	9.9	Superior temporal gyrus	No map
67.5	-35	18	9.6	Inferior parietal cortex	IPC (PF): 80% (assigned)
35	-55	-18	9.6	Fusiform gyrus	No map
47.5	-20	-9	9.5	Middle temporal gyrus	No map
40	-50	-21	9.3	Right fusiform gyrus	No map
50	-25	-3	9.2	Superior temporal gyrus	No map
55	-67.5	0	9.2	Middle temporal gyrus	hOC5 (V5): 30%
52.5	-75	0	9.1	Middle temporal gyrus	hOC5 (V5): 10%
57.5	-2.5	-12	9	Superior temporal gyrus	No map
45	-50	-24	9	Fusiform gyrus	No map
55	12.5	-15	9	Temporal pole	No map
47.5	-72.5	-15	8.9	Inferior occipital gyrus	No map
50	-30	0	8.7	Superior temporal gyrus	No map
45	-65	9	8.6	Middle temporal gyrus	hOC5 (V5): 30%
20	-2.5	-24	8.5	Hippocampus	EC: 50% (assigned)
50	7.5	-21	8.5	Temporal pole	No map
40	-62.5	-15	8.4	Fusiform gyrus	No map
67.5	-25	-3	8.4	Middle temporal gyrus	Te 3: 40% (assigned)
40	-50	-15	8.4	Fusiform gyrus	No map
55	-67.5	9	8.3	Middle temporal gyrus	hOC5 (V5): 20%
52.5	0	-18	8.2	Middle temporal gyrus	No map
50	-57.5	-18	8.1	Inferior temporal gyrus	No map
65	-52.5	9	7.9	Middle temporal gyrus	IPC (PGp): 10%
50	-57.5	9	7.9	Middle temporal gyrus	IPC (PGp): 10%
30	0	-27	7.9	Amygdala	LB: 70% (assigned)
47.5	-72.5	-3	7.8	Inferior temporal gyrus	No map
55	-27.5	9	7.8	Superior temporal gyrus	No map
50	-40	12	7.8	Superior temporal gyrus	No map
52.5	-70	-9	7.8	Inferior temporal gyrus	hOC5 (V5): 10%
32.5	-50	-12	7.8	Fusiform gyrus	No map
45	-80	-9	7.5	Inferior occipital gyrus	hOC4v (V4): 10%
40	-77.5	6	7.4	Middle occipital gyrus	No map
60	-52.5	3	7.4	Middle temporal gyrus	IPC (PGa): 10%
47.5	-32.5	12	7.3	Superior temporal gyrus	IPC (PFcm): 10%
25	-82.5	-12	7.3	Fusiform gyrus	hOC3v (V3): 80% (assigned)
50	15	-30	7.3	Medial temporal pole	No map
27.5	-65	-12	7.2	Fusiform gyrus	hOC4v (V4): 40% (assigned)
27.5	-80	-6	7	Fusiform gyrus	hOC3v (V3v): 40% (assigned)
42.5	-30	15	7	Superior temporal gyrus	OP 1: 40% (assigned)
25	-45	-15	7	Fusiform gyrus	No map
50	-47.5	9	6.9	Middle temporal gyrus	IPC (PGa): 10%
40	-57.5	3	6.7	Middle temporal region	No map
45	17.5	-33	6.7	Medial temporal pole	No map
45	-82.5	9	6.7	Right middle occipital gyrus	IPC (PGp): 20%
37.5	-85	18	6.5	Middle occipital gyrus	No map
37.5	0	-21	6.4	Temporal pole region	No map
25	10	-27	6.3	Parahippocampal gyrus	No map
30	-90	27	6.3	Superior occipital gyrus	Area 18: 10%
32.5	10	-33	6.2	Medial temporal pole	No map
37.5	7.5	-27	6.1	Temporal pole	No map
35	-75	-15	5.8	Fusiform gyrus	hOC4v (V4): 40% (assigned)
40	-72.5	24	5.7	Middle occipital gyrus	No map
17.5	-42.5	-6	5.6	Parahippocampal gyrus	SUB: 20%
27.5	2.5	-36	5.3	Hippocampus	EC: 80% (assigned)
-37.5	-52.5	-21	8.9	Fusiform gyrus	No map
-42.5	-45	-21	8.6	Fusiform gyrus	No map
-40	-40	-24	8.4	Fusiform gyrus	No map
-35	-65	-18	8.4	Fusiform gyrus	No map
-42.5	-57.5	-18	7.9	Fusiform gyrus	No map
-20	-57.5	-12	6.9	Linual gyrus	hOC4v (V4): 20%
-42.5	-80	-15	6.7	Fusiform gyrus	hOC4v (V4): 30%
-45	-80	-6	6.3	Inferior occipital gyrus	hOC4v (V4): 10%
-25	-45	-12	6.3	Fusiform gyrus	No map
-27.5	-50	-9	6	Fusiform gyrus	No map
-20	-37.5	-15	5.7	Fusiform gyrus	No map
-62.5	-40	6	8.6	Middle temporal gyrus	Te 3: 20%
-62.5	-32.5	12	8.3	Superior temporal gyrus	Te 3: 40% (assigned)
-57.5	-30	12	8.2	Superior temporal gyrus	OP 1: 40% (assigned)
-50	-77.5	9	8	Middle occipital gyrus	IPC (PGp): 20%
-50	-37.5	9	7.6	Middle temporal gyrus	No map
-60	-27.5	0	7.6	Middle temporal gyrus	No map
-57.5	-15	-9	7.5	Middle temporal gyrus	Te 3: 10%
-47.5	-35	21	7.3	Inferior parietal cortex	IPC (PFcm): 70% (assigned)
-52.5	-37.5	3	7.3	Middle temporal gyrus	No map
-62.5	-17.5	-6	7.3	Middle temporal gyrus	No map
-52.5	5	-21	7.2	Middle temporal gyrus	No map
-57.5	-2.5	-6	7.2	Superior temporal gyrus	No map
-57.5	-12.5	3	7	Superior temporal gyrus	Te 1.2: 40% (assigned)
-47.5	-40	3	7	Middle temporal gyrus	No map
-60	-7.5	-6	7	Middle temporal gyrus	Te 3: 10%
-55	-37.5	18	6.9	Inferior parietal cortex	IPC (PFcm): 40% (assigned)
-55	-62.5	12	6.7	Middle temporal gyrus	IPC (PGa): 10%
-47.5	-55	9	6.6	Middle temporal gyrus	No map
-50	12.5	-21	6.5	Temporal pole	No map
-55	7.5	-6	6.5	Temporal pole	No map
-55	-50	9	6.4	Middle temporal gyrus	No map
-45	-50	3	6.4	Middle temporal gyrus	No map
-52.5	-60	6	6.3	Middle temporal gyrus	No map
-50	-67.5	15	6.3	Middle temporal gyrus	IPC (PGp): 30%
-57.5	-40	24	6	Inferior parietal cortex	IPC (PF): 50% (assigned)
-55	-47.5	0	5.8	Middle temporal gyrus	No map
-62.5	-47.5	15	5.8	Superior temporal gyrus	IPC (PFm): 20%
-42.5	-75	15	5.7	Middle occipital gyrus	No map
-50	-5	-15	5.5	Middle temporal gyrus	No map
-42.5	-67.5	9	5.5	Middle temporal gyrus	hOC5 (V5): 10%
-47.5	-80	21	5.3	Inferior parietal cortex	IPC (PGp): 70% (assigned)
45	20	21	8.3	Inferior frontal gyrus	Area 45: 10%
-12.5	-75	-45	7.5	Cerebellum	No map
-17.5	-77.5	-39	7.4	Cerebellum	No map
55	32.5	0	7.2	Inferior frontal gyrus	Area 45: 50% (assigned)
50	35	9	6	Inferior frontal gyrus	Area 45: 40% (assigned)
55	27.5	-6	5.4	Inferior frontal gyrus	Area 45: 50% (assigned)
50	5	48	7	Precentral gyrus	Area 6: 10%
-22.5	-7.5	-18	6.9	Amygdala	LB: 90% (assigned)
-15	-7.5	-27	5.6	Hippocampus	EC: 90% (assigned)
12.5	-27.5	-3	6.8	Dorsal midbrain	No map
17.5	-20	-12	5.9	Hippocampus	SUB: 10%

**Fig 2:**
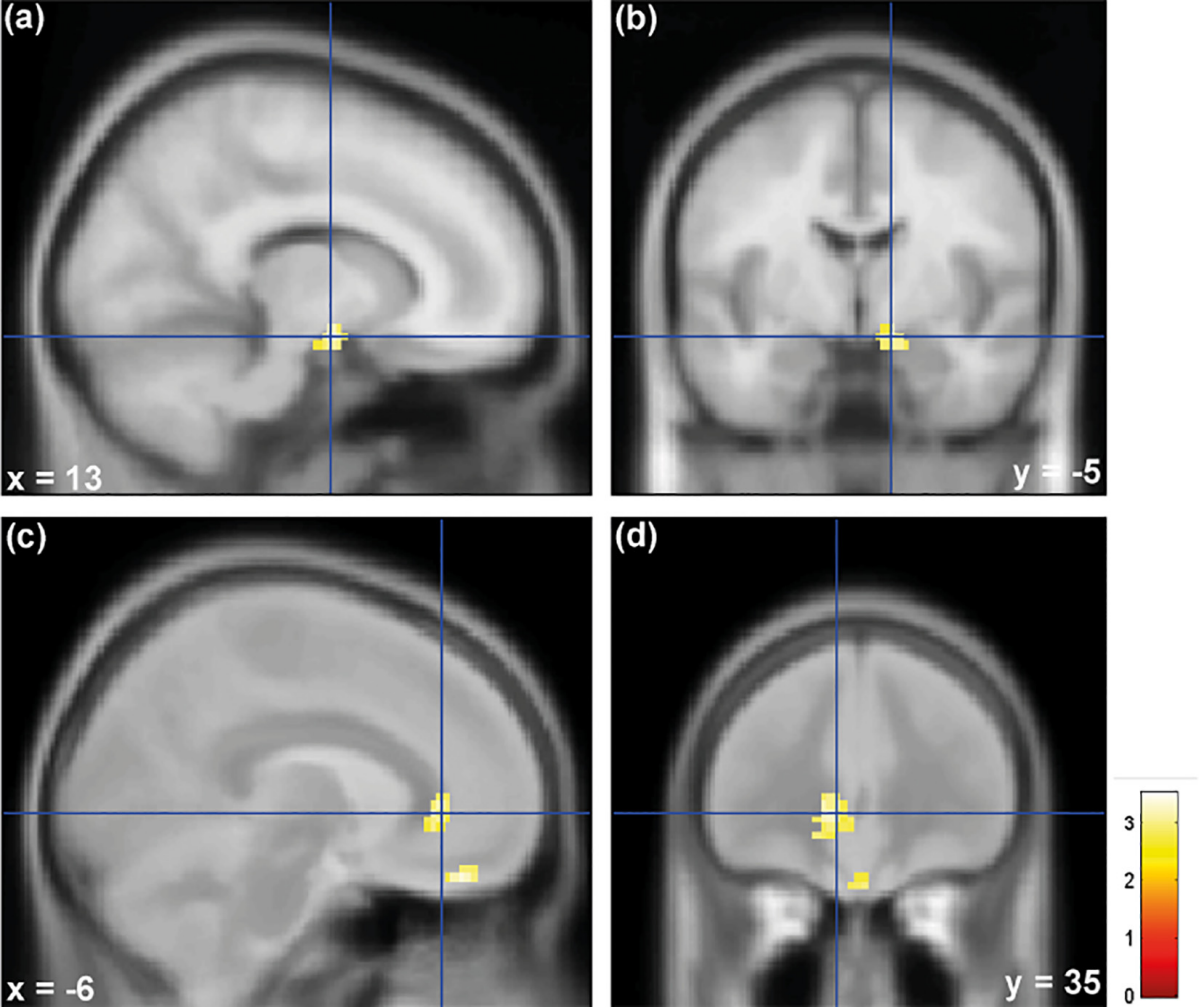
Shows that Individuals with high neuroticism showed stronger activation in the (a)(b) amygdala and (c)(d) subgenual anterior cingulate cortex (sgACC) when exposed to infant crying compared to individuals with lower neuroticism levels (p < 0.05, FWE small volume corrected). The median (neuroticism score: 1.67) served as the boundary between the high and the low neuroticism groups. A 10 mm radius sphere was placed in sgACC (MNI coordinate: 6/42/-16) and the amygdala (MNI coordinate: 22/-8/-12) based on previous findings that show the involvement of sgACC and amygdala in neuroticism [17]. The results are superimposed on the MNI-152 standard brain (SPM12).

Individuals with high neuroticism levels showed stronger activation in the amygdala and sgACC when exposed to infant crying compared to individuals with lower neuroticism levels (p < 0.05, FWE small volume correction, see Fig 2). Anatomical assignments were performed using a probabilistic anatomical atlas system [41,42].

### Psychological data

Neuroticism scores (M = 1.76, *SD* = 0.62) were in a normal range [43]. All subjects were right-handed according to the Edinburgh handedness questionnaire [34] (mean = 84.11,% SD = 12.69). Across the six blocks showing the video clip with crying infants, arousal decreased (b = –0.083, SE = 0.020, *p*<0.001), and irritation increased (b = 0.089, SE = 0.035, *p* = 0.012), whereas no significant trend was observed for valence (b = –0.003, SE = 0.018, *p* = 0.868). Individuals with increased neuroticism experienced more irritation (b = 0.460, SE = 0.218, *p* = 0.038, for linear relationship) and perceived infant crying as more unpleasant (valence ratings were more negative; b = –.281, SE = 0.121, *p* = 0.022) in comparison to individuals with lower neuroticism scores. Arousal was not related to neuroticism (b = .197, SE = 0.130, *p* = 0.132). We did not detect any interaction effects between time and neuroticism (*p*>.05 for all three outcome measures). To test for fatigue we presented at the end of the experiment a video clip that showed laughing infants (i.e., participants perceived another emotional category than during the habituation phase). We compared Cry_6 with Laugh_2 and found significant differences for all three psychological variables, with arousal and irritation exhibiting strongly reduced values (arousal, b = –0.871, SE = 0.177, *p*<0.001; irritation, b = –1.586, SE = 0.155, *p*<0.001), and valence exhibiting strongly increased values (b = 3.172, SE = 0.126, *p*<0.001) when showing a video clip with laughing rather than crying children. At the end of the experiment (block Laugh_2) psychological values for arousal and valence were not related to neuroticism (arousal: b = 0.069, SE = 0.227, t = 0.303, p = .763; valence: b = -0.243, SE = 0.139, t = -1.748, p = .083). Psychological values for irritation were significantly higher in subjects with higher neuroticism scores (b = 0.675, SE = 0.194, t = 3.475, p < .001).

### Physiological data

**Fig 3:**
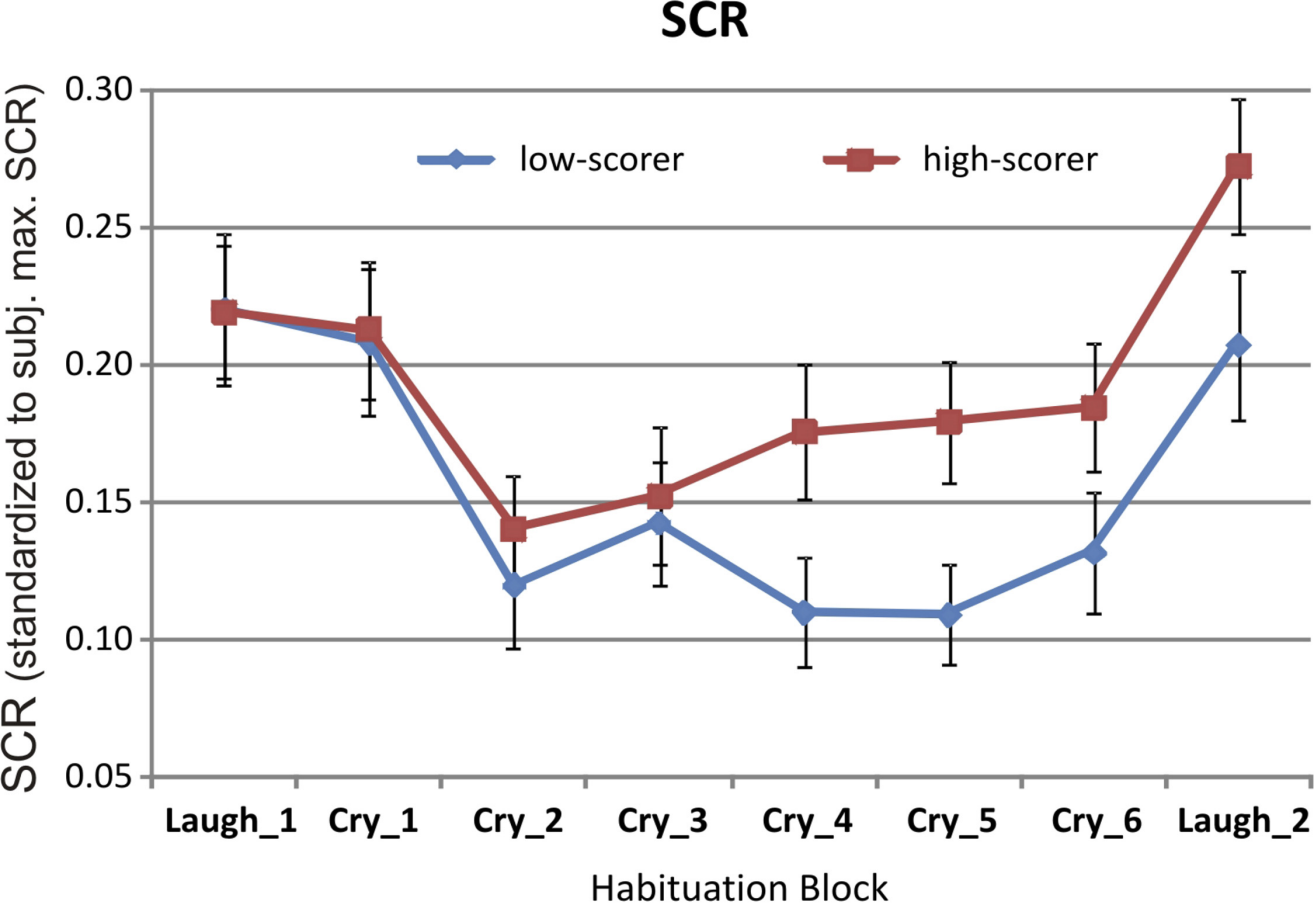
Illustrates time course of skin conductance responses (SCR, group means). For this illustration the median (neuroticism score: 1.67) served as the boundary between the high and the low neuroticism groups (please note: in the statistical model neuroticism was used as continuous variable). Five video clip presentations were averaged into one block, i.e. the first block summarizes five video clip presentations that showed laughing infants at the beginning of the fMRI experiment, the following six blocks summarize each five video clip presentations that showed crying children, and finally the last block summarizes five video clip presentations that showed laughing children at the end of the fMRI experiment. Women scoring higher on neuroticism showed overall stronger skin conductance responses to the ongoing exposure to film clips of crying infants. Error bars indicate standard errors.

SCR showed a curvilinear temporal trend over the six blocks of repeated presentation of crying children: SCR values first strongly decreased from the first to the second block but then remained more or less at the same level towards block 6 (linear time trend, b = –0.005, SE = 0.003, *p* = .118; quadratic time trend, b = 0.006, SE = 0.002, *p* < .001; Fig 3). Averaged across temporal blocks, SCR values showed a curvilinear relationship with participants’ neuroticism: SCR values increased from low- to relatively high neuroticism scores, but declined again to average values for highest neuroticism scores (linear trend, b = 0.022, SE = 0.014, *p* = 0.104; quadratic trend, b = –0.040, SE = 0.019, *p* = 0.034). Participants with the highest neuroticism values also displayed high CES-D values (see Fig B in S1 File). When adjusting the model for CES-D values, the relationship between neuroticism and SCR was still somewhat curvilinear but less so for women with high neuroticism values (*p* = 0.014 for linear trend, *p* = 0.047 for quadratic trend). CES-D values themselves had little effect on SCR values (*p* = 0.063 for linear relationship). The test for fatigue at the end of the experiment (comparing block Cry_6 with block Laugh_2) showed that SCRs were strongly increased when displaying a video clip with laughing rather than crying children at the end of the experiment (b = 0.069, SE = 0.016, *p*<0.001, Fig 3). At the end of the experiment (block Laugh_2) SCR values were not related to neuroticism (SCR: b = 0.025, SE = 0.023, t = 1.094, p = .277, see Fig 3).

## Discussion

At the neural level, this study found that infant crying activates the left and right inferior frontal gyrus (including Broca's area and Broca's homologue on the right hemisphere), the left anterior insula, left and right fusiform gyrus, hippocampus, amygdala, and superior temporal gyrus (all brain regions are listed in Table 1). Research shows that infant crying activates the amygdala in nonparental women [44]. Riem and colleagues found that women without children with insecure attachment showed increased amygdala activation when exposed to infant crying compared to women with secure attachment [27]. Recently, it has been shown that the administration of oxytocin (a neuropeptide that facilitates mother-infant bonding) to nonparental women reduced activation in amygdala and increased activation in the insula and in the inferior frontal gyrus [5]. The amygdala and the anterior insula are brain structures that have been associated with emotional responding [36,45]. Previous functional imaging studies have shown that the anterior insula plays are role in empathy [46] as well as during the perception of infant vocalizations [47]. Research shows that the amygdala is involved in face processing [48] and responsive to infant vocalizations [49]. A recent study found, the amygdala responds to human and computer-generated faces (avatar faces) similar and that the fusiform gyrus showed a greater response to human faces, suggesting that that fusiform gyrus may help the human brain to distinguish computer-generated faces from real faces [50]. The right inferior frontal gyrus may be involved in decoding emotional facial expressions [51,52]. A recent study suggests that the inferior frontal gyrus and superior temporal gyrus may play a role in affective prosody comprehension [53]. Furthermore, significant BOLD signal decrements (i.e., habituation) in response to a repeatedly presented video clip that showed crying infants were found in the fusiform gyrus, middle temporal gyrus, superior temporal gyrus, Broca’s homologue on the right hemisphere, (laterobasal) amygdala, and hippocampus (all brain regions are listed in Table 2). The left inferior frontal gyrus (including Broca`s area) did not show significant habituation (see Fig 1). Previous research shows that the (laterobasal) amygdala is preferentially activated in response to emotionally valenced stimuli and habituates in response to repeatedly presented human faces [23] and music [21]. A study by Britton and colleagues demonstrates that both the amygdala and fusiform gyrus habituate over time in response to emotional facial expressions [54]. Habituation has been considered to be a simple form of learning that allows humans to filter out irrelevant and focus selectively on important stimuli [32]. Researchers believe that habituation might be a prerequisite for other forms of learning [32].

Our results suggest that individual differences in neural processing of infant distress are related to differences in neuroticism. Individuals with high neuroticism levels showed stronger activation in the amygdala and in sgACC when exposed to infant crying compared to individuals with low neuroticism. Stronger activation in sgACC may indicate enhanced cognitive control in women scoring high in neuroticism when repeatedly exposed to infant distress. A recent study found that sgACC correlates positively with fear levels when participant chose to bring a live snake closer to the scanner. These findings suggest that sgACC plays an important role in cognitive control during the process of successfully overcoming a real-life stressful situation [20]. A study by Haas and colleagues found that neuroticism correlated positively with sgACC and amygdala activation during trials of high emotional conflict, compared with trials of low emotional conflict [17], which may reflect increased cognitive control in individuals with high neuroticism during an emotionally distressing task. Based on several theories suggesting people differ substantially in environmental sensitivity (for an integrative overview see reference [9]), we hypothesized that high-neuroticism individuals are more emotionally responsive and experience more negative emotions during the exposure to infant distress. In agreement with these hypotheses, we found that women with high neuroticism showed stronger amygdala activation and experienced more irritation, and perceived infant crying as more unpleasant in comparison to individuals with lower neuroticism scores. Arousal ratings, however, were not affected by neuroticism. Furthermore, individuals with high neuroticism demonstrated significantly greater SCRs in response to infant crying. These findings are consistent with recent research showing that elevated neuroticism is associated with increased physiological reactivity (e.g., electrodermal activity) during distress [14,55]. Taken together, individuals with high neuroticism showed stronger emotions during the repeated exposure to infant crying which is reflected by the fact that they showed stronger amygdala activation, greater SCRs, experienced more irritation, and perceived infant crying as more unpleasant in comparison to individuals with lower neuroticism scores. We argue that individuals with high neuroticism therefore exercise more cognitive control, which is reflected by the fact that they also displayed stronger sgACC activation. As mentioned above this brain region plays an important role in cognitive control during the process of successfully overcoming a stressful situation.

In contrast to our prediction we found no evidence that neuroticism impacts habituation on the neuronal, peripheral-physiological or affective-behavioral level.

Lin and McFatter suggested that infant crying may evoke two different emotional responses: distress and empathy [3]. In this study we found that infant crying evoked in all participants activation in the inferior frontal gyrus and the anterior insula. Riem and colleagues suggested that those brain regions may underlie empathy in response to infant crying [5]. A future study, however, is required to examine whether the activations we found in the inferior frontal gyrus and anterior insula in response to infant crying are associated with empathy. Our study found in agreement with Lin and Mc Fatter`s suggestion that individuals with high neuroticism experienced more negative emotions during the exposure to infant crying, indicating that they felt more distressed than individuals with low neuroticism. During the perception of *infant laughter* at the end of the experiment SCR values and psychological values for arousal and valence were not related to neuroticism, yet, psychological values for irritation were higher in subjects with higher neuroticism scores. This finding might be in agreement with previous research showing that increased neuroticism goes along with stronger emotional responses during “negative events” such as infant crying and also with heightened emotional responses during “positive events” such as infant laughter [8,15].

Some participants with high neuroticism scores who also obtained high scores on the CES-D [33], which assesses depressive symptoms, displayed relatively low skin conductance responses (see Fig B in S1 File). It has been previously shown that individuals with depressive symptoms may demonstrate suppressed SCR responses [56]. This has also been found in individuals with subsyndromal depression [57].

In the following paragraph potential limitations of this study are discussed and suggestions are made for future research. We tested for fatigue by presenting at the end of the experiment stimuli from a different emotion category (i.e., a video clip that showed laughing infants). This study found highly significant differences for all three variables of emotional reactivity (valence, arousal and irritation) as well as for the level of SCRs between the laughing and crying conditions. These findings are in agreement with the definition of habituation according to which response decrement results from repeated stimulation and does not involve sensory and motor fatigue [32]. A future study should investigate habitation effects during the repeated exposure to infant laughter in order to clarify whether the habituation effects we observed in this study during infant crying are specific or a more general habitation effect.

To reduce sources of variance not directly related to the study aims this sample was confined to healthy women. Women have been found to score higher than men on neuroticism [58] and show compared to men differences in brain activity in response to infant cries [29], underlining that generalization of our findings to men is an important topic for future research. Neuroticism scores in this study spanned the normal range [43] and hence results do not inform about clinical neuroticism. Whether the results generalize to clinical levels of neuroticism needs to be examined in future research. Furthermore, future studies may use the Highly Sensitive Person (HSP) scale by Aron & Aron [12] to test for individual differences in environmental sensitivity. The hypothesis for differences in environmental sensitivity was developed after data collection was completed. Hence, this study relied on neuroticism as a marker of environmental sensitivity (in [12], HSP was significantly associated with neuroticism with r = .41). As mentioned, research suggests that neuronal responses to infant crying may be modulated by parenting experiences [28]. Future studies may consider investigating neuronal, physiological, and affective habituation patterns in response to infant crying in mothers and fathers because neuroticism may impact parenting [59]. An interesting area for future research is (similarly to a study by Riem et al., [60]) to investigate amygdala-connectivity during the perception of infant crying.

In conclusion, the findings of this study show that individuals high in neuroticism are more emotionally responsive, experience more negative emotions, and may show enhanced cognitive control during the exposure to infant distress, which may impact infant-directed behavior. This study presents a valuable approach to investigate neuronal, physiological and emotional responses to infant crying by using simultaneous fMRI and SCR recordings and the assessment of emotional experience.

## Supporting Information

S1 File **Fig A. Illustration of the experiment design.** (A) State-anxiety was assessed in the scanner before (STAI before) and after the experiment (STAI after). (B) Females were *familiarized* with the experimental setting and a video clip showing laughing infants (LI) was presented five times. Subsequently, in order to assess *habituation*, a video clip that showed crying infants (CI) was presented 30 times. A crontrol movie was presented three times. In order to distinguish habituation from sensory and motor fatigue we tested for fatigue at the end of the experiment by presenting the video clip with laughing infants five times. Individuals rated valence, arousal, and irritation after every video clip presentation. (C) Each video clip consisted of a sequence of five laughing or five crying children with a total length of 15 s. **Fig B. Some participants with high neuroticism scores who obtained high scores on the Center for Epidemiologic Studies Depression Scale displayed low skin conductance responses.** The highest neuroticism scores are shown in squares. *X-axis*: Shows scores on the *Center for Epidemiologic Studies Depression Scale* (CES-D). *Y-axis*: Shows skin conductance responses (SCR, standardized to subject`s max SCR) during the exposure to infant crying.
(DOCX)
