# Supplementary material for: The Role of the Subgenual Anterior Cingulate Cortex and Amygdala in Environmental Sensitivity to Infant Crying
Source: PLoS One. 2016 Aug 25;11(8):e0161181. doi: 10.1371/journal.pone.0161181 (PMC4999060; doi:10.1371/journal.pone.0161181)
Supplement: S1 File — Fig A. Illustration of the experiment design. (A) State-anxiety was assessed in the scanner before (STAI before) and after the experiment (STAI after). (B) Females were familiarized with the experimental setting and a video clip showing laughing infants (LI) was presented five times. Subsequently, in order to assess habituation, a video clip that showed crying infants (CI) was presented 30 times. A crontrol movie was presented three times. In order to distinguish habituation from sensory and motor fatigue we tested for fatigue at the end of the experiment by presenting the video clip with laughing infants five times. Individuals rated valence, arousal, and irritation after every video clip presentation. (C) Each video clip consisted of a sequence of five laughing or five crying children with a total length of 15 s. Fig B. Some participants with high neuroticism scores who obtained high scores on the Center for Epidemiologic Studies Depression Scale displayed low skin conductance responses. The highest neuroticism scores are shown in squares. X-axis: Shows scores on the Center for Epidemiologic Studies Depression Scale (CES-D). Y-axis: Shows skin conductance responses (SCR, standardized to subject`s max SCR) during the exposure to infant crying. (DOCX) [file pone.0161181.s001.docx]

**Supporting Information**


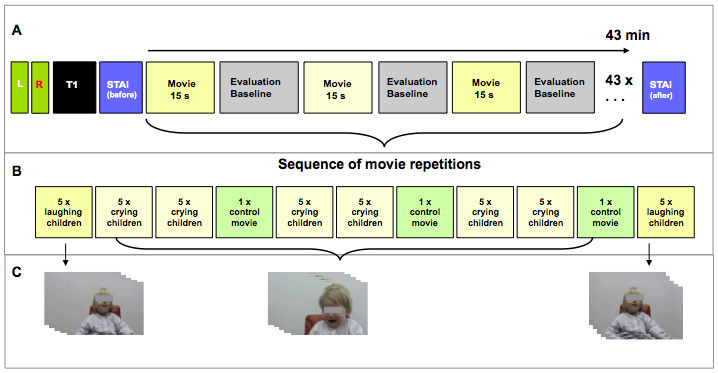


**Fig A. Illustration of the experiment design. A** State-anxiety was assessed in the scanner before (STAI before) and after the experiment (STAI after). **B** Females were *familiarized* with the experimental setting and a video clip showing laughing infants (LI) was presented five times. Subsequently, in order to assess *habituation,* a video clip that showed crying infants (CI) was presented 30 times. A crontrol movie was presented three times. In order to distinguish habituation from sensory and motor fatigue we tested for fatigue at the end of the experiment by presenting the video clip with laughing infants five times. Individuals rated valence, arousal, and irritation after every video clip presentation. **C** Each video clip consisted of a sequence of five laughing or five crying children with a total length of 15 s.

**Stimuli**

Ten typically developing infants and toddlers were recorded with a digital video camera while sitting in a child’s chair and expressing emotions (4 girls and 6 boys, mean age=16.6 months, range= 12-21 months). These video recordings were approved by the ethics committee of the University of Freiburg, Germany. Before participation, parents gave their written informed consent. Recorded video sequences of the children were edited using Ulead Media Studio Pro 8 (Ulead, Taiwan) and video clips of a sequence of five laughing (laughing infant video clip, LI) or five crying children (crying infant video clip, CI) were created with a total length of 15 seconds. An attention-control variant of the CI film was created by replacing the last child in the sequence with a repetition of the first child. The presentation order of individual children’s recordings within the stimuli for both conditions (LI and CI) was permuted over subjects to eliminate any systematic differences caused by the order of stimuli.

**
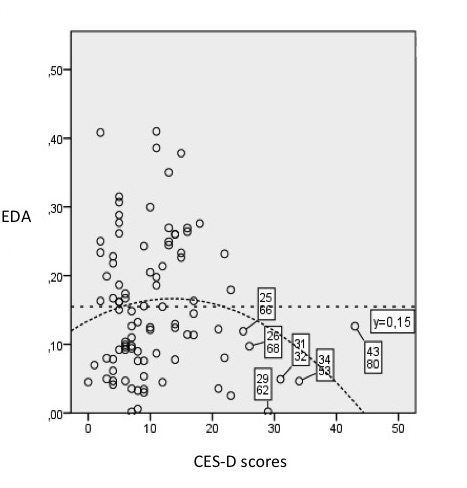
**

**Fig B. Some participants with high neuroticism scores who obtained high scores on the Center for Epidemiologic Studies Depression Scale displayed low skin conductance responses.** The highest neuroticism scores are shown in squares. *X-axis*: Shows scores on the *Center for* Epidemiologic Studies Depression Scale (CES-D). *Y-axis*: Shows skin conductance responses (SCR, standardized to subject`s max SCR) during the exposure to infant crying.

1. Radloff LS (1977) The CES-D scale: A self report depression scale for research in the general population’. Applied Psychological Measurement 1: 385-401.
